# Ghosts in the Machine. Interoceptive Modeling for Chronic Pain Treatment

**DOI:** 10.3389/fnins.2016.00314

**Published:** 2016-06-30

**Authors:** Daniele Di Lernia, Silvia Serino, Pietro Cipresso, Giuseppe Riva

**Affiliations:** ^1^Department of Psychology, Università Cattolica del Sacro CuoreMilan, Italy; ^2^Applied Technology for Neuro-Psychology Lab, IRCCS Istituto Auxologico ItalianoMilan, Italy

**Keywords:** chronic pain, interoception, interoceptive modeling, free energy, predictive coding

## Abstract

Pain is a complex and multidimensional perception, embodied in our daily experiences through interoceptive appraisal processes. The article reviews the recent literature about interoception along with predictive coding theories and tries to explain a missing link between the sense of the physiological condition of the entire body and the perception of pain in chronic conditions, which are characterized by interoceptive deficits. Understanding chronic pain from an interoceptive point of view allows us to better comprehend the multidimensional nature of this specific organic information, integrating the input of several sources from Gifford's Mature Organism Model to Melzack's neuromatrix. The article proposes the concept of residual interoceptive images (ghosts), to explain the diffuse multilevel nature of chronic pain perceptions. Lastly, we introduce a treatment concept, forged upon the possibility to modify the interoceptive chronic representation of pain through external input in a process that we call interoceptive modeling, with the ultimate goal of reducing pain in chronic subjects.

## Background

Human beings are embodied organisms, our bodies provide the substrate for a broad range of experiences that deeply intertwine emotions, feelings, physical and psychological conditions. For this very reason, the body constitutes a core element in human mental representations as well. Albeit its role in human cognition has always represented a challenge, in the past years the study of the inner representation of the body has become hectic thanks to the compelling work of Craig on the lamina I spinothalamocortical system that redefined the concept of interoception as the sense of the physiological condition of the entire organism (Craig, 2003).

Craig's work provided neuroanatomical evidence of a metacognitive matrix in the right anterior insula cortex (AIC) that integrates input from all the tissues through specific afferent primary fibers (Aδ and C), creating the perception of the “material self” that sustains subjective feelings and self-awareness (Craig, 2002, 2003, 2004, 2009). These inputs are collected from a wide range of physiological systems; afferent lines are activated in a graded manner by all sort of autonomic functions from temperature, to pain, from immune, hormonal, and cardiovascular activity, to touch, hunger and thirst, effectively creating a complex metacognitive map of all the active processes in the organism. AIC displays connections to the anterior cingulate cortex (ACC) that co-activates in several imaging studies (Reiman, 1997; Bartels and Zeki, 2000; Damasio et al., 2000; Blood and Zatorre, 2001) providing evidence that ACC incorporates the behavioral and motivational agent that co-participates to the emotional appraisal system.

The metarepresentations in the AIC are structured upon the integration of salience across all physiological afferent information at each moment of time and can be conceptualized as a series of “global emotional moments” (Craig, 2009) moving from the past to the anticipated future, in a self-aware stream of consciousness of cinemascopic “images” of the organism that creates our embodied self-perception. In particular, AIC seems to contain sub-sequential metarepresentations (in the range between seconds and subseconds) of all the physiological functions active in the organism, and the salience of a factor, in this cinemascopic representation within the interoceptive matrix, is assessed considering the significance of the factor for the optimal homeostasis and wellbeing of the organism (Jänig, 2006).

A second implication, strictly connected with the concept of sub-sequential metarepresentations of the bodily states across the time, is the necessary presence of anticipatory models within the interoceptive matrix. The main function of the serial set of representations of all interoceptive inputs across a finite period of time (Craig, 2009) is to provide a functional homeostatic control. Thus, this function requires “anticipatory global emotional moments” generated through an internal behavioral model composed by experiences and expectations, which creates adjustments in the system by comparing the anticipated prediction with the objective status of the body. Moreover, this specific point of view redefines the concept of emotional moments as subjective and flexible, considering that the anticipatory behavioral model can subsequently adjust the internal pattern in the interoceptive cortical system. This buffer, or comparator, serves as core apparatus in the predictive function of the interoceptive awareness (Craig, 2009), nonetheless the informational capacity for salience in the AIC must also be finite, thus a high rate of accumulation could fill-up the emotional buffer in a quite short time-frame.

Craig's proposal of interoceptive salience across time as an anticipatory global emotional moment, relates to the free energy principle (Friston, 2009) and process theories or corollaries like predictive coding. According to the free energy theory, an organism in order to optimize his functions must avoid surprising states, which are considered as incoherent predictions between the actual state of the organism and the expected condition. Within the homeostatic system, this approach suggests that global emotion anticipatory models must reduce the discrepancy between predictions of interoceptive status and actual homeostatic functioning, with the ultimate goal of keeping biological processes within specific functional bounds. Interestingly, in the formal setting of predictive coding and active inference, salience can also be considered as salience of sampling and it “can be defined operationally in terms of minimizing conditional uncertainty about perceptual representations” (Friston et al., 2012). This fits comfortably with the notion of anticipatory models that are trying to reduce the variability or uncertainty about homeostatic outcomes.

The idea that avoidance of unexpected events requires a predictive model is not new. Predictive models inside the interoceptive inference framework (Gu and FitzGerald, 2014) can in fact rely on decision making processes as an instrument to modulate the behavioral activation in order to promote a functional homeostasis, reframing Damasio's somatic marker hypothesis as an embodied predictive coding contest (Seth, 2014).

Optimal reduction of free energy can be achieved through two different processes, predictive/perceptual coding (Rao and Ballard, 1999; Friston, 2005) and active inference (Friston et al., 2011; Joffily and Coricelli, 2013). These two pathways propose an inverted sequence of activation, while predictive coding represents a change in the prediction to match the unexpected sensation, the active inference process excites autonomic reflects to create visceral feelings that fulfill top down predictions. Consequently, interoceptive predictions errors and their resolution actuated by the cortical matrix might contribute to the global experience, both from an emotional point of view and from a motivational point of view (Seth, 2013).

Recently, Barrett and Simmons (2015) proposed an Embodied Predictive Interoception Coding (EPIC) model that effortless integrates predictive coding theory with interoceptive studies about body perception. Among other interesting hypotheses, Barret identified three ways of minimizing prediction errors, along with active and perceptual inference, the authors suggested that the cognitive network can modulate the sensation through a shifting in the attention focus, actually resampling the input, modulating the gain of neurons that represent the salience of the incoming sensation (Barrett and Simmons, 2015). This idea finds support in the sampling and scrutinizing dimensions of the Mature Organism Model (MOM) (Gifford, 1998) along with the recent work of Talsma that considered attention as a form of predictive coding (Talsma, 2015).

Lastly, Ondobaka et al. (2015) hypothesized that the sensory afferent streams to the interoceptive cortex are structured in a hierarchical way. Different levels of disposition are therefore distributed in a coherent continuum that moves from abstract information (higher hierarchy) to visceral inputs (lower hierarchy), supposing that higher levels entail overly precise predictions about low level representations; thereby precluding a revision of high level (integrative) representations or beliefs—by precise interoceptive input. This perspective finds support in the compelling work of Edwards et al. that proposed a neurologically informed model of hierarchical Bayesian inference (Edwards et al., 2012). In this model, top-down prior predictions are strictly dependent on the relative precision of each hierarchical level. This precision is encoded by the synaptic gain (or post-synaptic responsiveness) in the upper layers of the cortex in those neurons that report prediction errors (Edwards et al., 2012; Adams et al., 2013), whereas a specific change in the synaptic gain (i.e., increase precision) can move the posterior beliefs toward the prior prediction. This perspective leads to an interesting conclusion according to which a primary failure of inference could be therefore characterized by the presence of overly precise priors, encoded by the synaptic gain in intermediate hierarchical levels (Edwards et al., 2012).

## Residual interoceptive ghosts across time. the R.I.G.T. hypothesis

Chronic pain is a complex topic. If acute pain fulfills an informative need aimed at the avoidance of the damage, chronic pain is purposeless (Allegri, 2015). Current definitions identify chronic pain as a specific pathology that persists for more than 3 months or beyond the expected time for healing (Merskey et al., 1994; Treede et al., 2015) but even though the nosology is clearly determined, the etiopathogenesis of chronic pain conditions is wide. Chronic pain can originate from pathologic conditions no longer solvable or beyond the regenerative capacities of the organism, as well from situations without any detectable organic dysregulation. In this complex contest, which considers pain as a multisensory and multidimensional perception, several theories propose different but integrative perspectives.

Melzack proposed that pain can be considered as an output of a multidimensional experience generated by specific neurosignatures that imposed themselves in a distributed neural network called neuromatrix (Melzack, 2005). This complex framework considers pain on different levels; specifically it can be generated by organic causes, such as an injury, but can also be triggered independently through the spontaneous activation of neurosignature patterns inside the neuromatrix, providing an elegant explanation for several chronic conditions without detectable organic alterations.

The Mature Organism Model (MOM) (Gifford, 1998) integrates several ideas and suggests that pain can be considered an integrative experience forged upon sampling, appraisal, scrutinizing, and response processes from a top-down perspective. The MOM proposed an embodied cognition of pain (Thacker, 2015) that sees pain as a perception derivate from a constant sampling and elaboration of the inner and outer environment in a predictive coding comparator, in close accordance to the free energy principle.

Although several chronic pain diseases (i.e., arthritis, low back pain, etc.) depend upon precise organic tissue damages that cannot be resolved, a specific set of psychopathological conditions (i.e., conversion disorders, somatoform disorders, etc.) elude a medical physiological diagnosis albeit they are characterized by chronic pain symptoms. From this point of view, Edwards et al. proposed another interesting perspective (Edwards et al., 2012), which reconciles Melzack's and Gifford's work. The authors suggested how pathologically precise priors are able to introduce a primary failure in perceptual inference, effectively shifting the posterior beliefs toward the predicted sensation, through attentional modulation of synaptic gain in the neurons that encode prediction errors on intermediate hierarchical levels, effectively generating symptoms such as pain.

**Figure 1:**
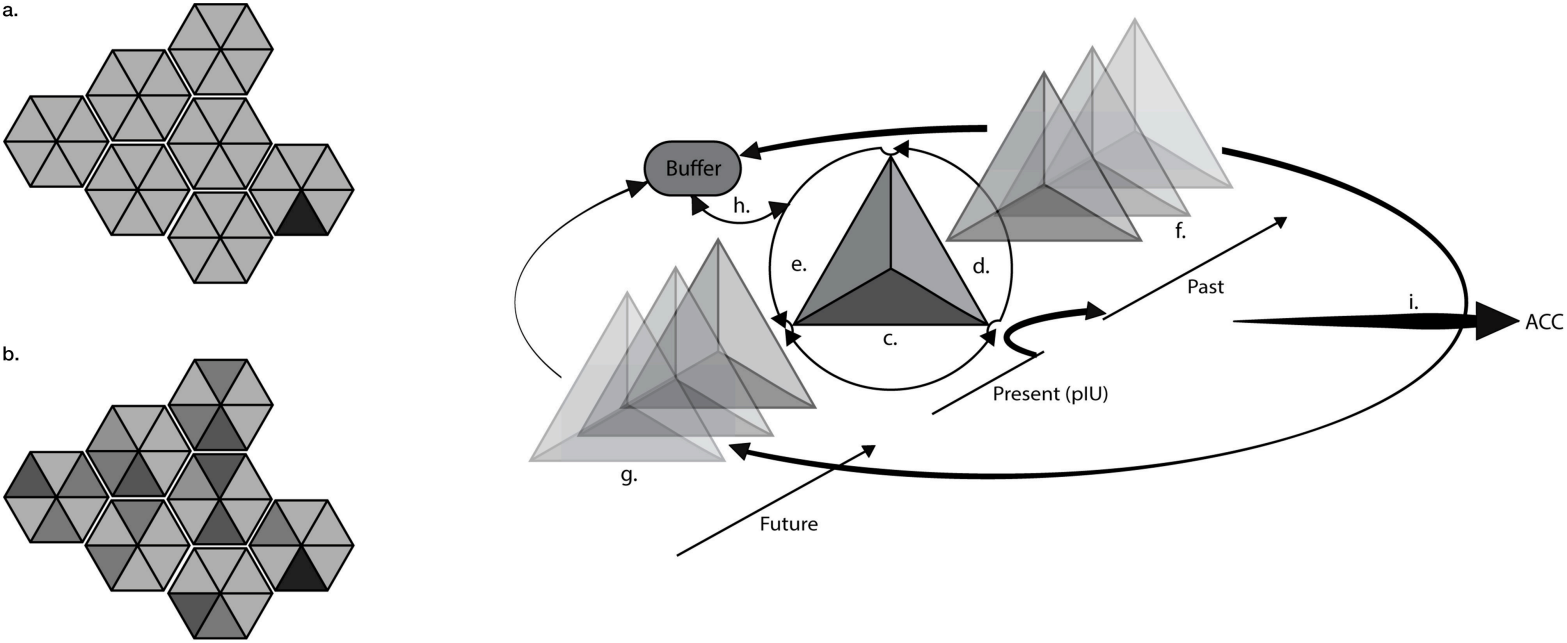
**Residual interoceptive ghosts across time**. The draws (left) represent the interoceptive matrix (IM) in which primary interoceptive units (triangles) are single pieces of the whole interoceptive landscape. The black triangle represents the primary interoceptive unit (pIU) connected to pain. In a healthy (a) subject, interoceptive units have a short life and are quickly updated and dispersed in a fluid bodily landscape. In chronic (b) conditions pIU is impressed in different dimensions creating residual interoceptive images (ghosts) across time, which easily fill-up the interoceptive buffer. Ghosts are represented by darker triangles; the shades of gray suggest different intensities and collocations across time. The drawing (right) represents the three-dimensional structure of interoceptive information, applied to chronic pain. The pIU is composed by the organic chronic information (c) weighed and compared with past stored patterns (d) and future iterations (e) about the state of the body. The pIU is stored in the past memories (f) and the past memories are used to forge the interoceptive prediction (g) in the future. Past memories, future predictions and actual state (pIU) of the body are confronted in the comparator (h) that also accesses the matrix to initialize compensatory responses. This cycle is dispersed (i) at different levels from the AIC to the ACC, according to Craig's global emotional moment. The residual past memories and future representations at all levels are detached from the original organic information thus they are, actually, residual images (ghosts) of the original input that represented the chronic pain information.

Merging these perspectives with Craig's work on the interoceptive matrix, and the predictive coding framework we hypothesize that chronic pain creates residual metacognitive representations inside the probabilistic (generative) model of the body (Talsma, 2015). If the interoceptive matrix collects physiological information from all the tissues of the body, inside this matrix we can necessarily find chronic interoceptive representations of pain (Figure 1). Three different dimensions compose these representations:

- a primary interoceptive pattern (Craig, 2009) that includes the organic chronic information weighed and compared with past stored patterns and future iterations about the state of the body. Interoceptive sensations do not exist only as raw organic information; these sensations are “largely prediction” (Barrett and Simmons, 2015) and thus they require a constant integration between expectations and past metacognitive bodily information. This dimension represents the present state of the body.
- a series of past representations of the primary interoceptive pattern that serve as base information to the predictive model within the buffer. This dimension represents the past state of the body.
- a series of future representations of the primary interoceptive pattern, forged upon past representations with the ultimate goal to predict the future state of the body and minimize the free energy (i.e., prediction error). This dimension represents the predictive future state of the body.

Although the primary interoceptive pattern represents the fundamental unit of the appraisal process, these three dimensions compose the interoceptive pathway that recursively develops altered chronic representations across time in upper layers through ascending integration, according to a hierarchical organization model (Edwards et al., 2012; Ondobaka et al., 2015). The iterative anticipation cycles between AIC and ACC, also integrate the behavioral agent through emotions, beliefs, environmental information, expectations and memories (Craig, 2002, 2003, 2009), creating a global emotional moment of the chronic pain, anchored in the past, in the present, and in the future and therefore resilient to changes.

We hypothesize that these three components create a “ghost” representation that is a residual image of the pain signature located in the interoceptive primary pattern, in the priors, and in the memories of past sensations. The “residual interoceptive ghosts across time” (R.I.G.T) hypothesis suggests multiple ghosts that can be identifying not only the interoceptive immanent level but also in the endless cinemascopic images composing the priors that anticipate the feelings of the body, as well in the past mnemonic metarepresentations of the state of the body.

The ghosts exist transversally on multiple levels in the past, in the present, and in the future, and “the interaction between a master comparator buffer and the time series of global emotional moments might be experienced introspectively as an “observer” that nonetheless cannot “see” itself” (Craig, 2009). It is therefore possible that sequential past ghost representations are stored as observer memories (i.e., “allocentric representations”) becoming resilient to updates provided by contrasting egocentric representations coming from perceptual inputs (Riva, 2014). This peculiar dimension enforces the perception of pain itself through a self-sustaining anticipatory loop in a distributed neural network. The ghost representations can also outlast the physiological injuries or, equally, they can anticipate the pain before the organic threshold (Harvie et al., 2015), creating auto-predictive “ghost patterns” inside the representation of the body.

The R.I.G.T. hypothesis integrates Melzack's neurosignature and the MOM proposal and it suggests a new contribution according to which chronic interoceptive patterns extend across time, in residual images (ghosts) not only in the present but also in the past and in the future. These distributed presences provide the self-sustained information for the primary anticipatory buffer comparator and at the same time extend themselves across the different layers that create the pathway under the global emotional moment (Craig, 2009). It is important to understand that ghosts exist for every physiological state because every state has a primary interoceptive representation reported at different levels in the brain and across time, nevertheless in non-pathological conditions ghosts have usually a brief shelf life. They are constantly updated, changed, and modified, creating a fluid interoceptive landscape as Craig suggested (Craig, 2009). Albeit this fluid nature, in pathological conditions ghosts become chronic, impressing themselves in a resilient way across multiple areas and multiple time dimensions in auto-sustained loops.

An ancillary hypothesis is that constant interoceptive information about chronic pain disrupts the interoceptive matrix on the three different levels, filling up the finite salience capacity of the buffer that serves as comparator (Craig, 2009) for the integration of the predictive metarepresentations. The “interoceptive saturation hypothesis” suggests that high accumulation rate of information fed by a chronic (constant) condition can saturate the matrix lowering the salience, or the precision according to Edwards et al. (2012), of other inputs. Interestingly, this provides an explanation to the low interoceptive accuracy (Garfinkel and Critchley, 2013; Garfinkel et al., 2015) in chronic pain subjects (Pollatos et al., 2011; Weiss et al., 2014; Duschek et al., 2015).

The buffer can be filled-up according to three different mechanisms of free energy principle: active and perceptual inference and shifting in attentional focus (i.e., redeploying precision to different streams of prediction errors). In chronic conditions, pain signals from the body can easily fill-up the buffer, the priors, and the mnemonic metarepresentation of past sensations, impairing the processing of other interoceptive inputs. Nevertheless, the connection between interoceptive accuracy (formerly known as “sensitivity”) and pain processing shows a complex nature. Recent evidence demonstrated that high interoceptive accuracy can predispose healthy subjects to an enhanced perception of acute pain and a decreased acute pain tolerance (Pollatos et al., 2012). Moreover, enhanced interoceptive accuracy seems to pose as major predictor to paradoxical pain experiences (Scheuren et al., 2014) in healthy subjects. To make sense of these results we have to consider that these studies explored the perception of acute pain that is neurologically and functionally different from chronic pain. As presented above, chronic pain is a specific pathology able to produce profound changes on both brain function and structure (Baliki et al., 2008; Geha et al., 2008; Tagliazucchi et al., 2010). Moreover, chronic pain shows a different informative value than acute pain: if chronic pain is purposeless (Allegri, 2015), acute pain has a functional value as protective response to damages. Therefore, it makes perfect sense that subjects with high interoceptive accuracy show an enhanced perception of pain because information that can help avoid damages must have high salience to preserve the homeostatic balance of the organism.

The “interoceptive saturation hypothesis” can also explain low interoceptive accuracy in other psychopathological conditions, such as anorexia nervosa (Pollatos et al., 2008). In these situations, the high accumulation rate of information that fill-up the buffer is provided by top-down processes that originate in the behavioral agent within the ACC, equally impairing the perception of bottom-up interoceptive input, probably through high precision priors (Edwards et al., 2012; Adams et al., 2013).

The saturation hypothesis presents a major implication, related to treatments. Although interoceptive training to improve accuracy can produce benefits (Schaefer et al., 2014) they may have limited applications and effectiveness because they do not suppress the interference from other inputs that fill-up the buffer. Event thought accuracy can be improved through interoceptive training, the limited size of the buffer poses an upper bound on the results that can be achieved, meaning that we cannot constantly improve a limited, or imprecise according to Edwards et al. (2012), function. We have to reduce or suppress the interference from other factors.

## A new idea for treatment: Interoceptive modeling

**Figure 2:**
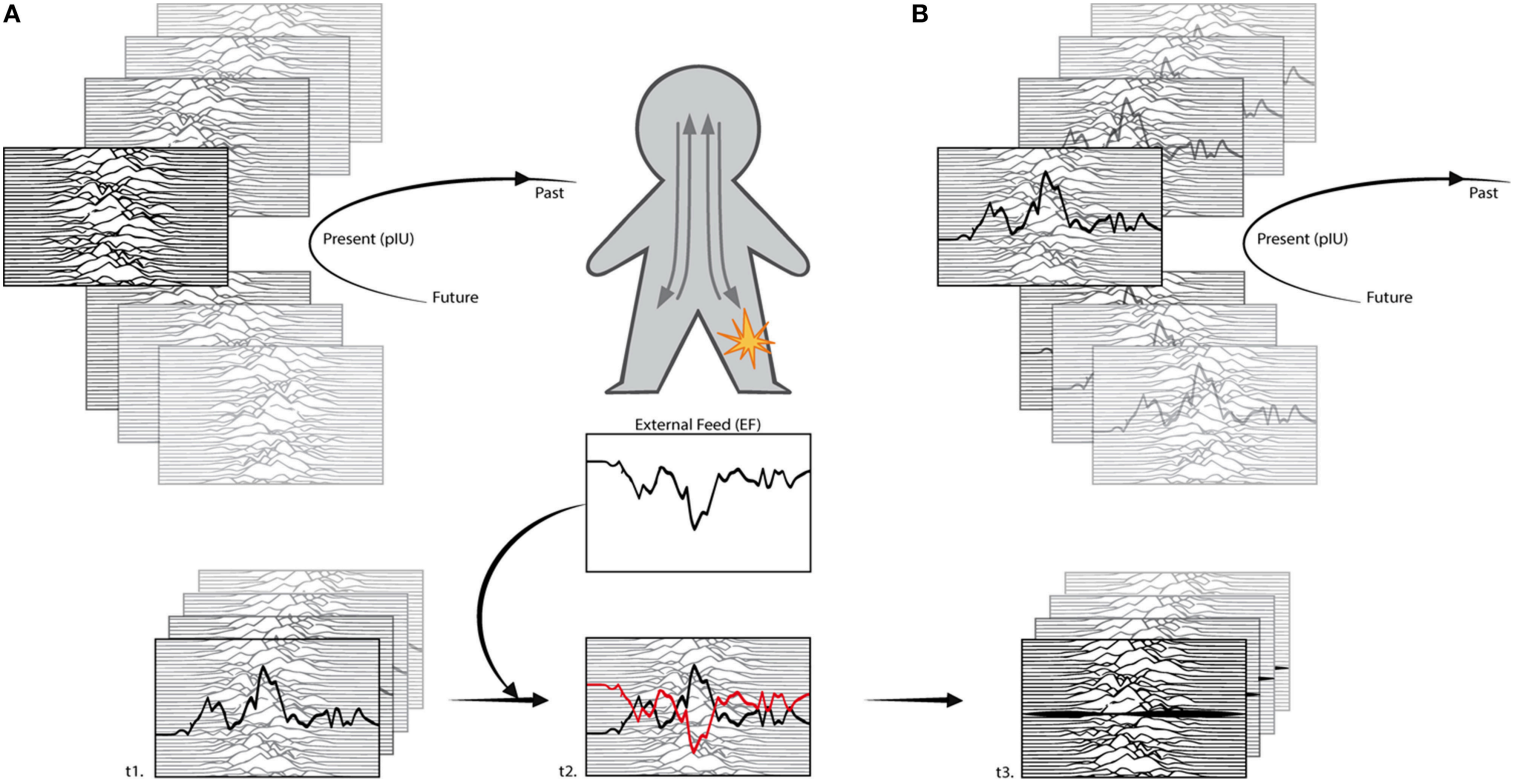
**Interoceptive modeling**. In Figure 1 we represented the interoceptive matrix (IM) as a set of static triangles, for convenience. Nevertheless, IM is fluid and dynamic, thus it can be better represented through a series of waves. In a healthy **(A)** subject, the landscape constantly changes while in a chronic pain **(B)** subject the signature of the pain is resiliently distributed across time, disrupting the access to other interoceptive information (fading background waves). Interoceptive modeling applies to a chronic pain subject (t1) and proposes to feed an external input (EF) to compensate (t2) the interoceptive primary representation of chronic pain. The concept is similar to counterphase modulation of sound waves, where two waves cancel each other (t3). To avoid prediction errors, the procedure will start mimicking the chronic interoceptive pattern and slowing modeling the feed through counterphase information. IM adapted from The Cambridge Encyclopedia of Astronomy.

According to our hypotheses, chronic pain is a resilient multicomposed perception that floods the interoceptive buffer with constant input, lowering subject's ability to access his own interoceptive information. The multidimensional nature of chronic pain defines the complexity of pain management treatments because they fail to compensate the distributed nature of these perceptions across time. To address the issue we propose the idea of interoceptive modeling (Figure 2), as a technique to feed interoceptive information through external (exteroceptive) input, forcing alterations in the interoceptive matrix.

Considering that “interoceptive sensation is largely prediction” (Barrett and Simmons, 2015), the core concept of interoceptive modeling is to update the priors from outside the body, through an exteroceptive feed of interoceptive information, rewriting the interoceptive primary representation and letting this modified pattern propagate in the global emotional moments, altering present, past, and future representations. This update can be considered a variant of perceptual inference because internal models are modified, nevertheless through an iterative loop the system will subsequentially promotes an active inference adaptation, because of constant updates emerging from the comparison between predictions and the actual state of the body.

Interoceptive modeling aims at altering the dimension of pain perception (priors) and the interoceptive signature created by a long lasting chronic condition, actively rewriting ghosts inside the distributed network. Nevertheless, we hypothesize that this kind of modeling will also produce physiological compensatory responses, considering that previous evidence suggested that interoceptive alterations can reflect on the body through autonomic pathways (Moseley et al., 2008; Barnsley et al., 2011; Tsakiris et al., 2011).

From a technical point of view, interoceptive modeling requires to superimpose external symbolic interoceptive information to compensate the interoceptive dysfunctional patterns. This concept entails two inalienable preconditions: that the subject is malleable to external input to determine his own interoceptive information, and that the external input must not violate the probabilistic representation of the interoceptive pattern.

Tsakiris specifically explored the “active modulatory role of interoception on the experience of the body from outside” (Tsakiris et al., 2011), providing evidence that subjects with low interoceptive accuracy are more susceptible to integrate exteroceptive input to determine information about their own body. At the same time, the work of Tsakiris proved that external information can alter the perception of the body both on the behavioral and on the autonomic level. Although interferences in the homeostatic regulation caused by a flow of exteroceptive information have already been demonstrated in previous studies (Moseley et al., 2008) the work of Tsakiris has been the first to identify the interoceptive system as predictive factor for malleability in the body representation. Specifically, Tsakiris suggested that subjects with low access to interoceptive information may rely more on exteroceptive input to determine their own body condition. Contrary, subjects with high interoceptive accuracy, who have access to more precise and stable information about their own body, could be more resilient to external influences. These findings set an important bound to the applicability of interoceptive modeling for treatment, such as only subjects with low interoceptive accuracy may be suitable for external manipulation of interoceptive patterns. Fortunately, chronic pain along with several different psychopathological conditions, showed a consistent pattern of low interoceptive accuracy, providing an innovative opportunity for interoceptive modeling treatments.

About the second precondition, Talsma advocated that multisensory integration depends upon a coherent internal mental representation that is a metacognitive merging of top-down and bottom-up processes (Talsma, 2015). The author suggested that a simple mismatch between information internally represented together, could lead to impaired performances because incongruent multisensory modalities violate the consistency of the model leading to increasing prediction errors. Contrary, congruent inputs are able to provide a consistent match to the internal representation, reducing the prediction error and increasing the consistency of the metacognitive representation (Talsma, 2015) also on interoceptive level. Other authors (Tsakiris et al., 2011) suggested that multisensory integration requires a coherent representation of the body, posing the multisensory congruency on different levels (visual, anatomical, spatial, postural etc.) as a core element of a successful merging. These evidence suggested that, to be effective, interoceptive modeling must feed information that can be consistently integrated in the interoceptive matrix, without excessively violating the internal consistency to avoid substantial prediction errors that will compromise the effectiveness of the treatment.

Interoceptive modeling is forged upon this framework, and it aims at projecting complementary interoceptive information that overrides chronic pain representations on the diffuse neural network. The process would feed exteroceptive information in subjects with low interoceptive accuracy to update interoceptive patterns connected to the chronic pain. Since chronic pain subjects with low accuracy rely on external input for interoceptive information, iterative discrepancies between the external feed and the interoceptive pattern can be resolved only by updating the internal model on the subordinate interoceptive level. This process will overwrite the mental representations of pain (ghosts) creating permanent alterations both at neurological level and at cognitive level. Furthermore, the possibility to elicit autonomic responses through active inference (Moseley et al., 2008; Barnsley et al., 2011) also suggests possible effects on physiological levels based upon the fact that interoceptive matrix not only work as store box for information but it usually also contains autonomic response patterns.

## Practical implications for treatment and challenges

As far as we know, interoceptive modeling treatments have never been theorized neither developed from a practical point of view. Nevertheless, although several implications and challenges need to be addressed, some previous studies support the concept of interoceptive modulation through external input.

Augmented reality (AR) or immersive virtual reality (VR) can offer instruments to promote interoceptive modeling, as Aspell demonstrated in a recent study. The author provided evidence that a subject can use exteroceptive information about the heart rate to modulate the perception of the body effectively shifting the sense of ownership and incorporation. The results suggested that interoceptive information fed by exteroceptive sources can influence the perception of the body (Aspell et al., 2013). Aspell experiment also proved that the body can use exteroceptive information to determine internal interoceptive properties; moreover the experiment demonstrated that subjects can use visual information of interoceptive representations to modulate the interoceptive matrix. This provides evidence that interceptive information can be represented in different forms, i.e., heartbeat has been represented as silhouette outside the body and nevertheless it was able to tap in the material self of the subject, creating a sense of ownership and altered tactile acuity, directly supporting the possibility to use visual and exteroceptive information to modulate the interoceptive representations. Another example is the work of Serino et al. (2016) that used exteroceptive VR input to provide an update of the internal representation of the body through a proprioceptive modeling in healthy subjects. Moreover, the VR approach to interoceptive modeling will reduce prediction errors leading to an increased sense of presence because in virtual environments perceptual inference can diminish the density of interoceptive signals, through salient external inputs that foster acceptance of exteroceptive information as “one's own embodied state” (Farb et al., 2015) creating an update of the simulation map.

If virtual or augmented reality can provide technical instruments for interoceptive modeling, one of the major challenges that must be resolved is the virtual representation of the interoceptive pattern associate to the chronic pain and its consequently complementary modelization that will serve as overwriting information. Nonetheless, working with pain simplifies the task to gather the interoceptive signature of the perception because several qualitative scales collect morphological and dimensional aspects of pain, along with its sensory characteristics. Among others, the Iconic Pain Assessment Tool, recently revisited in a web-based tool (Lalloo et al., 2014) provided 15 somatic markers (Damasio, 1996; Damasio et al., 2000) that describe different type of sensations connected to pain. These markers are unique and each one represents the symbolic translation of a specific type of pain, thus they can be utilized to precisely track the interoceptive pattern of pain sensation and to build its virtual representation and its complementary overwriting feed.

Albeit the ultimate goal of interoceptive modeling is to update the internal models through a process that resembles perceptual inference, the treatment must not violate the internal coherence of the bodily representation (Talsma, 2015) avoiding pervasive prediction errors that can impair the process. Possible impairments in the treatment are related to the fact that subjects can oppose the external feed due to unsustainable mismatching conflicts or worse, a feed that violates the probabilistic representation of the body can be nonetheless integrated creating pervasive prediction errors leading to stressful autonomic responses (Farb et al., 2015).

To avoid violations in the probabilistic representation of the body, interoceptive modeling envisages two phases of the treatment. In the “mimicking phase” the external feed must mimic the interoceptive information we want to compensate (i.e., chronic pain); the mimicking must be internally coherent with the representation of the body, reproducing the characteristics of the chronic interoceptive pain information and fostering multisensory congruency (Tsakiris et al., 2011; Zeller et al., 2015) upon different dimensions. In fact, multisensory congruency during the mimicking phase will not only integrate pain somatic markers collected via qualitative scales, but it will also integrate multisensory information through spatial, visual, anatomical, and other physiological inputs.

The mimicking phase aims at pairing the external feed with the internal representation of the body, helping subjects to accept the external feed as “one's own embodied state” (Farb et al., 2015) due to the fact that low interoceptive accuracy fosters multisensory integration of external inputs, especially when these inputs are coherent (Talsma, 2015) with the interoceptive representation.

After the integration of the external feed, without prediction errors or autonomic stressful responses, the treatment will introduce the “modeling phase” to manipulate internal representations connected to chronic pain. Modeling aims at altering the external inputs through counterphase information in an adjusted process that will slowly change the feed to counterbalance the initial mimicked representation. To avoid congruency violations in the probabilistic representation of the body, the modeling phase could introduce alterations in a modulatory incremental method that resembles the concepts of “titration” and “pendulation” in the practice of Somatic Experiencing (Levine, 1976, 1997, 2010). Specifically, titration refers to a process that sequentially introduces graduated changes, allowing the interoceptive system to adapt without triggering a cascade of stressful autonomic responses; while pendulation refers to a process that fosters oscillations between activation and deactivation phases to improve the balance of the autonomic system, re-framing the interoceptive representations (Payne et al., 2015). Interoceptive modeling will use a similar method to conjugate incremental systematic alterations (titration) of the external feed in an oscillatory way (pendulation) to tap in the interoceptive ghost representations without violating the coherence of the representation of the body (Zeller et al., 2015). Moreover, while some information related to the chronic pain (i.e., somatic makers that represent the pain and its intensity) will be changed, other information (i.e., spatial, anatomical, etc.) will remain congruent with the probabilistic representation of the body, allowing the treatment to introduce updates in the internal models without eliciting pervasive violations.

## Conclusion and future directions

In the present paper, we identified a three dimensional conceptualization of chronic pain, hypothesizing that an overflow of interoceptive information, predictive inferences, and mnemonic representations can produce residual interoceptive images (ghosts) across time. These ghosts, which could therefore rely on high precision priors encoded in the synaptic gain of prediction error neurons (Edwards et al., 2012; Adams et al., 2013), can saturate the buffer inside the interoceptive cortex, leading to a diminished interoceptive accuracy.

Evidence suggested that subjects with low interoceptive accuracy are open to utilize exteroceptive input to modulate their own interoceptive landscape, both on a behavioral/cognitive level and an autonomic level as well. This particular condition allowed us to propose a new idea for treatments forged upon interoceptive modeling, that is the use of exteroceptive input to modulate interoceptive patterns, through overwriting informational feeds. Interoceptive modeling requires different conditions; the predisposition of the subject to incorporate exteroceptive information, a coherent representation of these information that must not violate the internal interoceptive coherence, a set of instruments able to feed information, and a precise modelization of the information and their compensative overwriting inputs.

In this regard, Farb et al. (2015) suggested that utilizing exteroceptive input to substitute interoceptive information to reduce prediction errors may create dysfunctional patterns. To sustain this affirmation the author referred to evidence from the rubber hand illusion (RHI) studies that found decreased skin perfusion and temperature (Moseley et al., 2008) along with increased stress hormone release (Barnsley et al., 2011) in the resting arm. Addressing the evidence proposed by Farb, we have to consider that every kind of violation can instantly elicit prediction errors, fostering autonomic dysregulations and impairing the results (Wallace et al., 2004; Kording et al., 2007) of a possible treatment. From this point of view, switching a hand in the RHI represents a huge violation of the conservatory representation of the body. Thus, we suggest that this kind of illusion actually increases prediction errors inside the network (i.e., mismatch between actual proprioceptive location and perceived location) leading to dysfunctional patterns, in a procedure fundamentally different from interoceptive modeling.

RHI is a very peculiar type of process that violates the basic principles of our proposed idea. Specifically, interoceptive modeling aims at not disrupting the conservative probabilistic representation of the body (Talsma, 2015) stored in the interoceptive matrix. As explained, the mimicking phase and the modeling phase aim at reducing violations toward the interoceptive representation, thus interoceptive modeling can hypothetically update internal models without inducing pervasive prediction errors or violations in the representation of the body.

Future directions are open to several possibilities; chronic pain treatments forged upon interoceptive modeling can provide an innovative and effective option for a disease that affects 1 in 5 adults in Europe (Breivik et al., 2006). Nevertheless, interoceptive modeling is not limited only to chronic pain. Every organic function is represented inside the interoceptive matrix, thus we can hypothesize that several physiological dysregulations (hormonal, immunological etc.) operate in the same manner creating an interference that extend across multiple dimensions in the distributed predictive network. Therefore, understanding how interoceptive modeling works can bring insight useful to develop treatments for a variety of conditions, well beyond chronic pain.

## Author contributions

DD developed the hypotheses presented along with the final draft of the manuscript. SS, PC, and GR supervised the rationale and the scientific contributions. All authors read and approved the final manuscript.

### Conflict of interest statement

The authors declare that the research was conducted in the absence of any commercial or financial relationships that could be construed as a potential conflict of interest.
